# In vivo haploid induction leads to increased frequency of twin-embryo and abnormal fertilization in maize

**DOI:** 10.1186/s12870-018-1422-2

**Published:** 2018-11-29

**Authors:** Liwei Liu, Wei Li, Chenxu Liu, Baojian Chen, Xiaolong Tian, Chen Chen, Jinlong Li, Shaojiang Chen

**Affiliations:** College of Agronomy and Biotechnology, China Agricultural University, Beijing, China

**Keywords:** Twin-embryo, In vivo haploid induction, Haploid induction rate, Twin-embryo kernel rate, Flow cytometry

## Abstract

**Background:**

In vivo haploid induction (HI) based on *Stock6*-derived inducer lines has been the most prevalent means of producing haploids. Nevertheless, the biological mechanism of HI is not fully understood, the twin-embryo kernels had been found during haploid induction, which may provide potential evidence for the abnormal double fertilization during HI.

**Results:**

We investigated twin-embryo frequency in progenies of different haploid inducers. Results reveal that increasing the HI potential significantly improved the frequency of twin-embryo kernels. Compared with the average twin-embryo kernel frequency (average frequency = 0.07%) among progenies pollinated by the haploid inducer line CAUHOI, the frequency of twin-embryo was improved to 0.16% in progenies pollinated by the haploid inducer line CAU5. This result was further confirmed by pollinating single hybrid ND5598 with four haploid inducers possessing differentiated HIRs, where twin-embryo frequency was highly correlated with HIR. Among 237 twin-embryo kernels, we identified 30 haploid twin-embryo kernels (12.66%), a frequency which was much greater than the average HI rate for three other inducer lines (frequency range 2–10%). In addition, aneuploids, occurred at high frequency (8 in 41 twin plants). This level of aneuploidy provides new insight into the abnormal double fertilization during HI. Moreover, we observed differences in growth rate between twin plants in the field, as 4.22% of the twin plants grew at a significantly different rate. Both simple sequence repeats markers (SSR) and 3072 SNP-chip genotyping results revealed that > 90% of the twin plants shared the same origin, and the growth difference could be attributed to aneuploidy, competition for nutrients, and possible hormone regulation.

**Conclusion:**

These results demonstrate that an enhanced HI ability can increase twin-embryo kernel frequency, and high frequency of both haploid twin-embryo kernels and aneuploidy observed in this research give us new insights to understand the mechanism of both HI and abnormal embryogenesis.

**Electronic supplementary material:**

The online version of this article (10.1186/s12870-018-1422-2) contains supplementary material, which is available to authorized users.

## Background

Fertilization and embryogenesis in higher plants are complex and tightly regulated processes. Polyembryony is an abnormal phenomenon that reported in certain species of flowering plants [1]. Spontaneous twinning is widespread in Arabidopsis, and many polyembryonic mutants have been identified. Mutations in the gene *TWIN* could establish a differentiated structure from the transformation of suspensor cells early in embryogenesis and lead to polyembryony finally. *twn*1 mutants could also produce additional embryos via suspensor transformation. While the *twn*2 mutants give rise to multiple embryos through abnormally differentiated suspensor cells [2–4]. Polyembryos observed in Arabidopsis *bim1* (which encodes a BES interacting Myc-like protein1) embryonic patterning mutants do not result from suspensor cell division, which suggests a multiple fertilization event or premature zygote cleavage [5]. In rice, the poly-embryo promoting gene (*OsPE*) was cloned by insertion of a T-DNA*/Ds* from a fertile mutant [6]. In further studies, Paul et al. reported that multiple embryos in the *OsPE* mutant are a consequence of sequential proliferation and cleavage of the zygotic embryos [7]. The spontaneous development of multiple embryos in maize was originally described by Schrenk [8]. Pesev et al. studied the possibility of breeding twin-embryo lines from a synthetic maize population which can significantly increase both protein and oil content compares to single embryo kernels [9]. The frequency of twin-embryo kernels varied from 2.1 to 25.3%, with an average of 11.8%. Another analysis on the twin-embryo phenomenon of maize inbred line VIR17 showed that two types of twin-embryos including both suspensorial embryony and typical cleavage of zygotic proembryo occur spontaneously, typical cleavage can also be induced by treatment of developing caryopses with 2, 4-dichlorophenoxyacetic acid (commonly known as 2, 4-D) after pollination [10].

Naturally occurring in vivo HI in maize is a rare phenomenon first reported in 1959, i.e., that haploids occur stably regardless of genetic background—when pollinated by the line *Stock6* [11]. The subsequent development of maize haploid inducer lines with a higher haploid induction rate (HIR) allowed breeders to generate haploids efficiently [12–14]. Consequently, in vivo HI has become the preferred mean of producing maize haploids [15, 16]. As the result, *Stock6* and its derived lines were termed “haploid inducers”. During the double-fertilization process with haploid inducers as male parents, most seeds develop with normal endosperm and embryos, with ploidy levels of 3n and 2n, respectively. Except for a certain proportion of haploids, i.e., with ploidy levels of 3n for endosperm and n for embryo [11, 17], some abnormal kernels accompany haploid production; which include those with aborted embryos or an empty pericarp with different degrees of endosperm abortion and increased heterofertilization [16–20]. In addition, in a study conducted in 1966, that 49 twin-embryo kernels were observed in 49,903 kernels derived from combinations with haploid inducers, which implied the possible relationship between twin-embryony and haploid induction [21]. However, owing to the low frequency of twin-embryo kernels and the limitation of molecular biology methods at the time, these authors could present little evidence upon the relationship between haploid induction and increased number of twin-embryo kernels. Later, Li et al. used different hybrids in crosses with two haploid inducers and obtained 26 pairs of twin seedlings, with which they employed morphological observation and molecular analysis to publish their preliminary findings for these seedlings [22]. The simple sequence repeats (SSR) marker genotypes for the twin seedlings were identical, although some of them varied with respect to agronomic performance. Qiu et al. [18] also found that twin embryos could be produced during HI and obtained results similar to those of Li et al. with respect to the genotypes and phenotypes of the twin seedlings [22].

Recently, the gene underlying HI was independently reported by three research groups in different journals. The consensus result was that a 4-bp insertion in exon 4 of the gene GRMZM2G471240 was found to cause a frame shift and loss of function which finally triggered HI [23–25]. However, the biological mechanism by which HI and concomitant abnormal kernels occurs in maize remains unclear. In this study, we carried out a detailed analysis of twin-embryo production during in vivo HI. The objectives were to explore the law of twin-embryo kernel rate (TEKR) during fertilization with different haploid inducers and to explore the mechanism by which twin embryos are generated during HI, which could also help clarify the mechanism of HI.

## Results

### Maize haploid inducers increase TEKR

In our first trial that included 17 different maternal inbred lines pollinated with two haploid inducers, we found only 60 twin-embryo kernels (Additional file 1) in 60,659 seeds. This extremely low TEKR (range 0–0.28%, average 0.08%) did not allow us to establish a definitive connection between a maternal heterotic group and twin-embryo kernel frequency. Among the 17 inbred lines, the three with the highest TEKR were 4F1, GY923, and Dan360, which belong to the combined group of Lancaster and Lvda Red Cob group.

We further analyzed the effect of the male parent on TEKR. As shown in Table 1, no twin-embryo kernels were found in self-pollinated ears of the tested inbred lines. In the ears pollinated by the inducer line CAUHOI, the TEKR ranged from 0 to 0.35% (average, 0.07%). In the progenies derived from the high HIR inducer line CAU5, the TEKR ranged from 0 to 0.33% (average, TEKR 0.16%). Although the average HIR for CAU5 was greater than that of CAUHOI, the difference in TEKR between CAU5 and CAUHOI was not significant (Student’s test) under sample size in this trail.

**Table 1:** Comparison of two haploid inducer lines, CAUHOI and CAU5, for haploid induction rate and twin-embryo rate

Male parent	Tested kernels	TEKR (%)		HIR (%)	
		Mean	Range	Mean	Range
CAUHOI	43,025	0.07 a	0–0.35	4.88 b^†^	0.9–11.86
CAU5	25,153	0.16 a	0–0.33	9.62 a	6.54–15.75
selfed	19,961	0	NA	0	NA

*HIR* haploid induction rate, *HIR (%)* (number of haploids/total number of induced seeds) × 100%, *TEKR* Rate of twin-embryo kernel rate. *TEKR (%)* (number of twin-embryo kernels/total number of induced seeds) × 100%

^†^Values followed by the same lowercase letter are not significantly different at *p* < 0.05

We next pollinated a single hybrid ND5598 with four haploid inducers (Table 2) for which the HIR varied from ~ 2 to ~ 10%. The HIR for CAU2, CAU5, CAU^wx^, and CAUHOI, as determined with hybrid ND5598, was 10.27, 9.42, 5.33, and 2.29% respectively; similarly, the corresponding TEKR values were 0.12%, 0.11%, 0.08%, and 0.02%, respectively. The HIR and TEKR showed high positive correlation with a coefficient of 0.97 (*p* < 0.05). Combining the phenomenon fo potential increased TEKR by haploid inducers in aforementioned trail, we thus concluded that the HI correlated with significantly greater numbers of twin-embryo kernels.

**Table 2:** HIR and TEKR in maize hybrid ND5598 pollinated by 4 haploid inducers

Female parent	Male parent	N^a^	HIR (%)	TEKR (%)
5598	CAU2	7637	10.27	0.12
	CAU5	2855	9.42	0.11
	CAU^wx^	8769	5.33	0.08
	CAUHOI	8208	2.29	0.02

^a^The total number of induced seeds

### Classification of twin-embryo kernels by morphology, color, and size of the two embryos

**Fig. 1:**
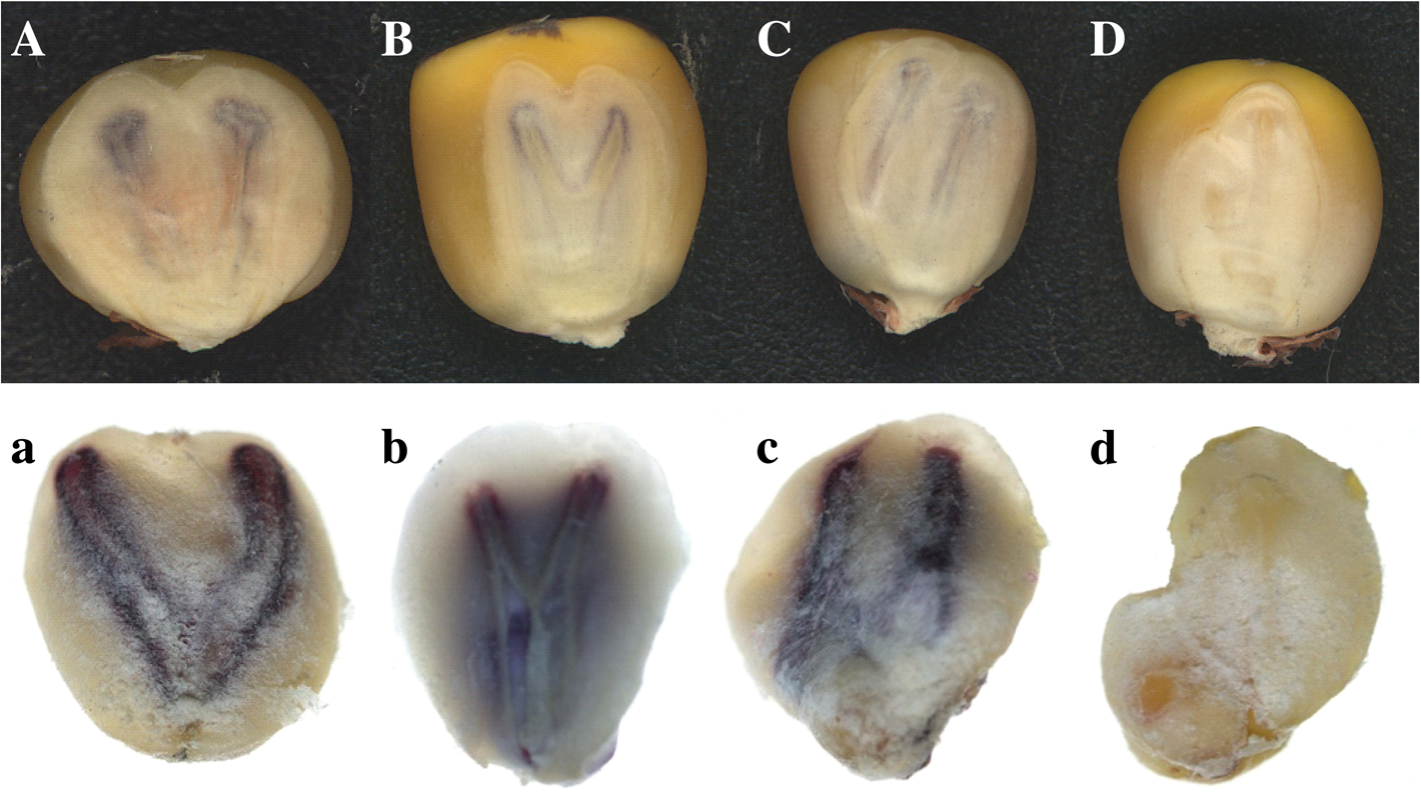
Classification based on the phenotype of twin-embryo kernels. A-D were type V, type Y, type II and uncertain type twin-embryo kernels. a-d were corresponding phenotype after embryo separation

For all the 237 twin-embryo kernels we acquired in the aforementioned experiment, fine classification was conducted according to embryo morphology, germ pigmentation, and embryo size of the twin-embryo kernels. Each unique type of twin-embryo kernel was counted (Table 3). As shown in Fig. 1, four structural types could be distinguished, namely type V, type Y, type II, and “uncertain”. For type V, the two germs diverge from the base, resembling the letter “V” (Fig. 1A). Similarly, the two germs of the type Y were divided at the middle of the embryonic axis (Fig. 1B). For type II, the two embryos were parallel to the embryonic axis and separate from one another (Fig. 1C). The twin embryos with an irregular shape did not fit any of the aforementioned types and were categorized as “uncertain”. As shown in Fig. 1, four structural types could be distinguished, namely type V (47.7% of the total), type Y (39.2%), type II (3.0%), and “uncertain” (10.1%) (Table 3).

**Table 3:** Numbers of different types of twin-embryo kernels

	Structure				Ploidy		Size^a^	
Classification	Type V	Type Y	Type II	Uncertain	Diploid	Haploid	Type A	Type B
Number	113	93	7	24	207	30	178	59

^a^Type I embryos were the same size between two plantules; type II embryos were of different size

**Fig. 2:**
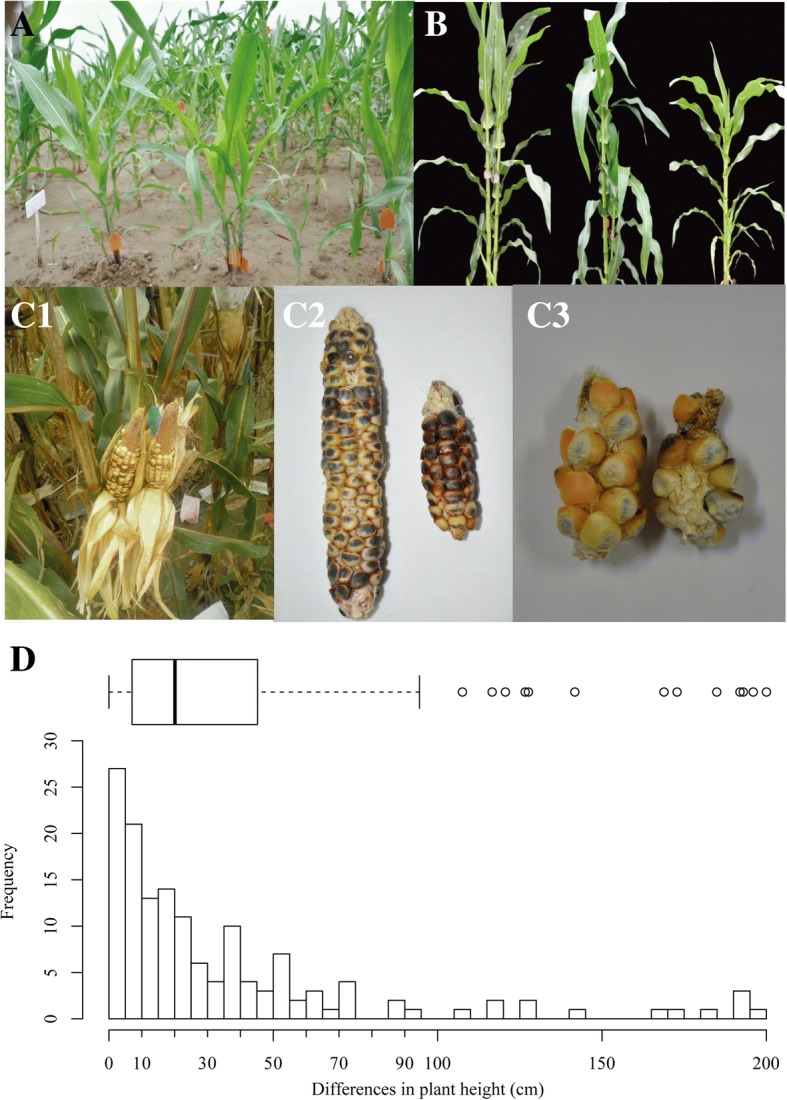
Field performance of twin embryo plants. A Twin plants in seedling stage. B PHD type I, II, and III twin plants in the field. C1-C3 Ear performance of three PHD types. D Variation of plant height difference between twin plants

**Fig. 3:**
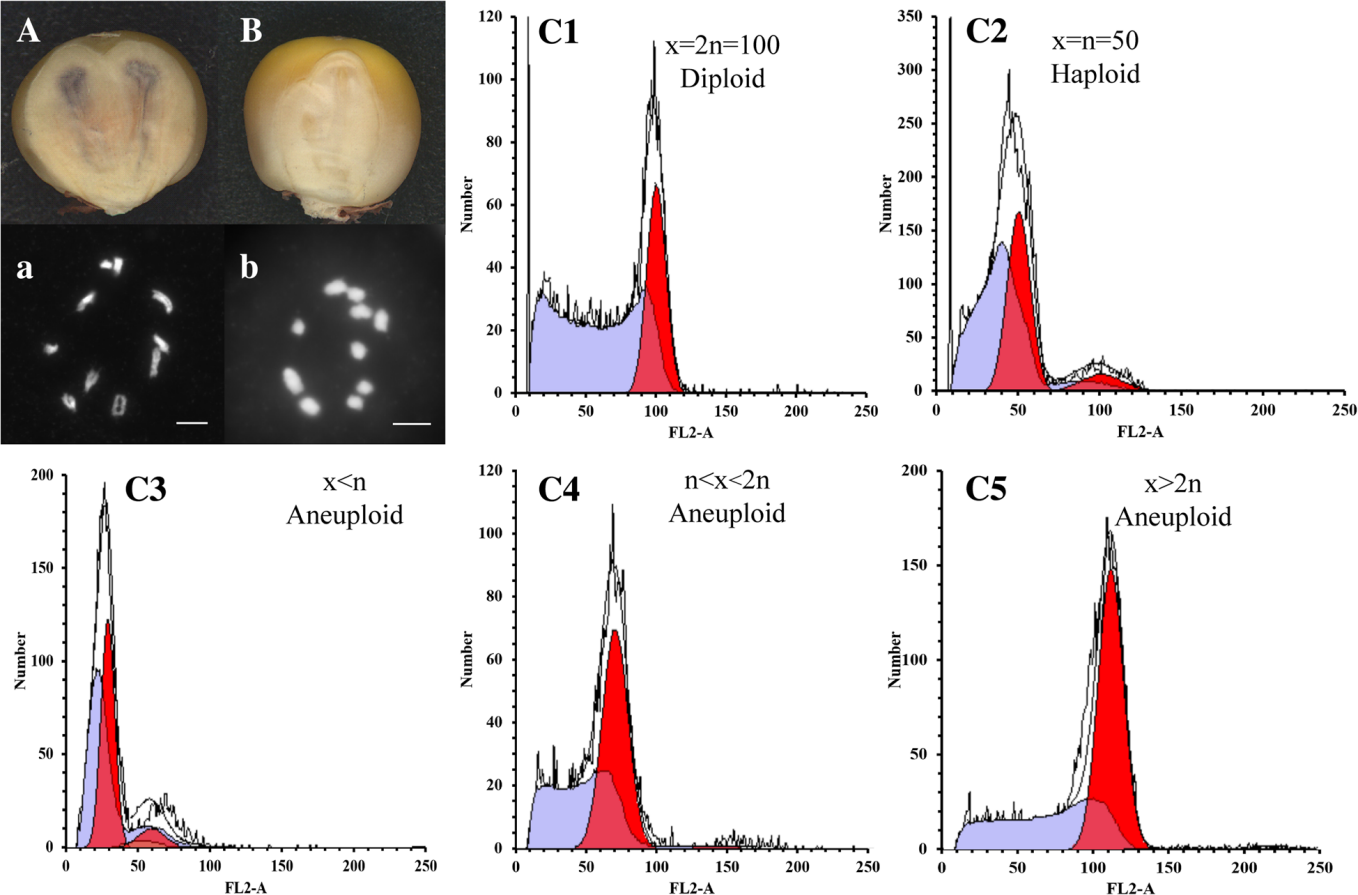
Ploidy determinations based on chromosome number and flow cytometry. A-B were diploid twin-embryo kernel and haploid twin-embryo kernel, respectively. a-b were chromosome number of corresponding diploid and haploid twin-embryo kernel. C1-C5 were flow cytometry result of diploids (C1), haploids (C2), and three kinds of aneuploids (C3, C4 and C5). Y axis is the number of cells detected, x axis represents for the fluorescent light area (FL2-A)

According to *R1-nj* pigmentation of the scutellum, the twin-embryo kernels could be classified as either purple or colorless (i.e., crossed hybrid, putative haploid) (Fig. 2A, B). Of 237 twin-embryo kernels, 207 were putative diploids with two purple embryos and 30 were putative haploids with two colorless embryos. To verify the ploidy level, chromosome number counting was performed for these putative twin-embryo haploids and diploids. The chromosome number was 20 for the putative diploid kernels (Fig. 3A, a), whereas it was 10 for the putative haploid kernels (Fig. 3B, b); this was consistent with the results obtained according to marker *R1-nj* and reconfirmed the accuracy of *R1-nj* identification system for twin-embryo kernels. The ratio of haploid twin-embryo was thus 30/237 × 100 = 12.66%, which was much higher than the average HIR. In addition, we compared the size of two plantules from each twin-embryo kernel and found that 75% of them were equidimensional, which we defined as type A, whereas the remaining 25% differed in size and were defined as type B (Table 3).

### Phenotypic and genetic variation between twin plants derived from twin-embryo kernels

Except for the twin-embryo kernels used for kernel anatomic analysis, of the 182 twin-embryo kernels that were germinated and grown to the seedling stage, 146 twin pairs survived to maturity. The individuals of each pair had the same shape at the early stages, but plant height varied as they grew (Fig. 3A, B). We categorized these twin plants according to plant height difference (PHD; see Fig. 3D) between the two individuals. As shown in the boxplot, except for 13 outliers (9% of the total), all others differed substantially with respect to plant height, and the PHD between individuals from most of the twin embryos was less than 100 cm. Based on the PHD for each of the individuals, we classified the twin-embryo plants into three types, namely PHD type I, II, and III. For type I, plant height for individuals in a pair varied by 0 to 20 cm, and their height remained essentially constant until harvest; the ears of each individual in this group were normal, and were either large (Fig. 3, C1) or small (Fig. 3, C3). For type II, however, the differences in height were quite large, and the harvested ears were correspondingly quite different in size (Fig. 3, C2). For type III, the smaller individual generally failed to grow to maturity, and for those plants that did mature, no ears were harvested owing to their small stature. The ratio of each type were PHD type I (49%), II (42%), and III (9.0%; 13 outliers). For these twin plants with large PHD, 41 pairs were chosen for flow cytometry analysis. Of these, 20 were diploid twin plants (Fig. 2, C1) and 13 were haploid twin plants (Fig. 2, C2 ). Interestingly, we detected 8 twin plants with aneuploidy plant, including 5 aneuploid with ploidy level between n and 2n (Fig. 2, C4), 2 aneuploids with ploidy level more than 2n (Fig. 2C5) and 1 aneuploid with ploidy level less than n (Fig. 2, C3), these aneuploids imply abnormal double fertilization and chromosome behavior during haploid induction.

### Genotypes of the twin plants

**Fig. 4:**
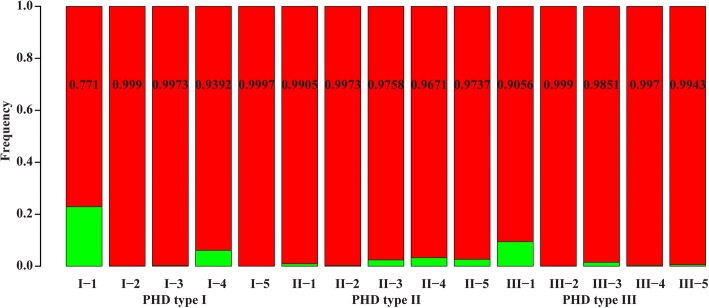
Genotyping results (SNPs) for plants from twin-embryo kernels. Red for the number of same genotype between two twin plants and green for different genotype between twin plants. Percentage of the same genotype is inside the red bar

Genotyping each of the 50 twin-embryo pairs with 30 SSR markers did not reveal any genetic differences between individuals—even among the three PHD categories. However, considering that the maize genome is so large that 30 molecular markers may not be sufficient to detect variants, we used the MaizeSNP3K Chip, containing 3072 SNP markers, for further genotyping. For this experiment, we chose 15 pairs of twin plants for genotyping—five pairs from each of the three PHD groups. Many genomic differences were found between the pairs of twin seedlings using the SNP-chip (Fig. 4). Nevertheless, polymorphism was greatest for twin pair I-1 from PHD type I, a group in which plant height varied little; the percentage of identical marker genotypes was only 77.1% for this pair of twin plants. The remaining 93% (14 of 15) of the twin plants showed > 90% identical marker genotypes. The variable loci were evenly distributed across the genome, and no continuous blocks of loci were detected in any of the twin-embryo plants.

## Discussion

### Production of twin-embryo kernels during HI

Production of twin-embryo kernels has been reported in several plant species. In maize, the double-embryo phenomenon was first reported in 1894 and then confirmed by subsequent research [1, 9, 10, 18, 22, 26, 27]. These investigators also identified other kernel abnormalities, including haploidy; haploid twin plants occur only rarely, however, and there was no distinct difference between normal kernels and twin-embryo kernels. Consequently, it has been difficult to study the mechanism or regulation of twin-embryo kernels. The discovery of the ancestral inducer line *Stock6*, which triggers 3.23% haploids in progenies, provided a new approach for acquiring maize haploids [11]. Owing to the huge utilization potential of doubled haploids (DH) in maize breeding programs in recent decades, many new inducers with higher HIR have been developed for DH breeding [12, 14, 28, 29]. The twin-embryo phenomenon during HI was first described by Sarkar and Coe in 1966, nevertheless little evidence on the relationship between HIR and TEKR was presented [21]. Our previous research confirmed the phenomenon of high frequency of twin-embryo kernels in maize ears pollinated by inducers [22], although it was difficult to determine whether the effect was attributable to haploid induction ability. In our present study, we designed an experiment to verify the phenomenon in a large population using different haploid inducers. We calculated the average TEKR of 17 inbred lines pollinated by both CAU5 and CAUHOI, and the results demonstrated possible improvement of TEKR in combination with CAU5. This effect also occurred in some specific materials such as Dan360 and Ye8112 (Additional file 2). This result was fruther verified by pollination of hybrid ND5598 with the four inducers CAU5, CAU^wx^, CAU2 and CAUHOI, which revealed a strong correlation between HIR and TEKR. Thus, we conclude that high HI ability can significantly improve the frequency of twin-embryo occurrence. Owing to the low frequency of TEKR, however, our results were insufficient to establish any definitive relationship among female parents.

### Fine classification of twin-embryo kernels

Studies on phenomenon of polyembryony in angiosperms have mainly focused on twin seedlings, including their classification and analysis of their variation. Here, we extended our previous observations to investigate the fusion of the two embryos based on the specific characteristics of the maize embryo. We found that 87% (206/237) of the observed twin embryos shared the same germ axis, including types V and Y. These twin embryos were inferred to arise mainly from the cleavage of the young embryo. Although haploid-diploid twin-embryo kernels have been reported [21], this kind of twin-embryo kernel was not found in our current or previous researches [22]. More importantly, an analysis on ploidy of the two germs from individual twin-embryo kernels revealed that 30 haploid-haploid kernels were found, which account for ~ 12.6% of all twin-embryo kernels. The frequency was higher than the average HIR of three inducer lines used in this research (CAU5, ~ 10%; CAU^wx^, ~ 5%; CAUHOI, ~ 2%;). These results demonstrate that, compared with diploids, haploid young embryos are more likely to undergo cleavage to yield twin-embryo kernels.

Except for diploid twin plants, aneuploidy in twin-embryo kernels occurred frequently in our present study, including aneuploids n < x < 2n, x > 2n, and x < n. Based on the phenotype of the twin plants in the field, we classified these twin plants according to their PHD; for most of the twin plants, the PHD was < 20 cm, although a moderate proportion of plants still had a large PHD. Aneuploidy reportedly affects plant growth in both Arabidopsis and maize [30, 31]; indeed, we observed frequent aneuploidy in twin germs, implying its important contribution to differences in growth. On the other hand, we did not detect huge genomic differences when genotyping was based on either SSR or SNP-chip; consequently, genetic differences could be ruled out. Apart from genetic factors, plant growth is also affected by the nutrient supply and interactions with the environment [32]. During the germination stage of twin-embryo kernels, the two germs compete for limited nutrients, and thus we may infer that competition for nutrients during either germ development or plant growth in the field contributed to our observed PHDs of twin plants. Among the 237 twin-embryo kernels we observed, 59 varied in size between the two germs. Differences between germs certainly will lead to a PHD. In addition, phytohormones especially 3-indoleacetic acid, play important roles in regulating growth and development [33, 34]. Most of the twin plants were type Y and type V, with connection parts, which may play an important role in communication between twin plants, similar to the tiller phenomenon in maize [35]. It is possible that the growth of small plants is inhibited owing to apical dominance.

### The mechanism underlying the production of different types of twin embryos

Although recent research demonstrated that loss of function of the gene encoding patatin-like phospholipase triggers HI [23–25], the mechanism by which *Stock6*-derived inducers trigger HI has not been fully elucidated. Evidence points to the abnormal development of pollen or its fertilization [16]. Except for haploids, an increase in HI ability can improve the frequency of embryo-aborted kernels, endosperm aborted kernels, and heterofertilized kernels [16–20], indicating that the genes or loci that contribute to HIR work against either normal double fertilization or early stage of development process. The observed increase in TEKR in combination with inducers with a high HIR could also be attributed to abnormal development. Two hypotheses, namely single fertilization and chromosome elimination have been posited to explain the mechanism of HI; there is supporting evidence for both hypotheses, such as the chromosome segments detected in haploids [36–38] and two differentiated sperm or differences in sperm mobility [39, 40].

Our current data reveal a high frequency of haploid-aneuploid twin-embryo kernels and diploid-aneuploid twin-embryo kernels (8 were aneuploid in 40 twin-embryo kernels). Considering that most twin-embryo kernels were type V and type Y and share the same origin from a single zygote, these haploid-aneuploid and diploid-aneuploid kernels could possibly be derived from zygotes that had undergone chromosome elimination; that is, if chromosome elimination was completed before embryo cleavage, the seeds would develop into haploid twin-embryo kernels. A continuation of chromosome elimination after cleavage of the young embryo would result in aneuploidy. Our results offer a new point of view that chromosome elimination may not occur immediately after pollination but rather occurs gradually during cell division. This is consistent with previous research that chromosome elimination is completed within 7 days after pollination [37]. Besides, as the special phenomenon in embryogenesis, twin-embryo showed high value for reproduction biology study. Taken together all these lines of evidence suggest that twin-embryos kernels found during in vivo haploid induction gives a new perspective to understand maternal haploid induction in maize.

## Conclusion

In this research, we demonstrated the effect of HIR on improving twin-embryo frequency during HI. Additionally, we conducted an in-depth classification of twin-embryo kernels that revealed a high frequency of young embryo cleavage. Moreover, high frequency of aneuploids were observed. These aneuploids give us a new perspective to understand the chromosome elimination process after HI.

## Methods

### Plant materials

#### Induction of twin-embryo kernels in diverse maternal inbred lines

To systematically evaluate the frequency of twin-embryo kernels, 17 elite maize inbred lines (Additional file 3) from different genetic backgrounds (heterotic groups) were used as female parents in crosses with the inducer lines to generate twin-embryo kernels. The five groups [41] are designated as Reid Yellow Dent (Reid), Lancaster Sure Crop (Lancaster), P group, Tang Sipingtou, and Lvda Red Cob group. Among these groups of inbred lines, Yu87–1 and Qi319 are from the P group, Longkang11, BY815, Ji846, GY923, 4F1, and Mo17 are from Lancaster, Dan360, Ye107 and Lv28 are from the Lvda Red Cob group, Zheng58, B73, Ye8112, Tie7922, and Ye478 are from Reid, and Jing24 is from Tang Sipingtou. The two inducer lines CAUHOI [36] and CAU5 [16] with distinct HIRs of ~ 2% and 10%, respectively, were used as the male parents.

Hand pollinations were performed with haploid inducers to produce twin-embryo kernels in maize as described [22]. The experiment was conducted in July 2012 at the Shangzhuang Experimental Station (39°56′N, 116°20′E) of China Agricultural University in Beijing, China. Ears harvested were air-dried. Putative haploid kernels can be identified on the ear using marker *R1-nj*, which yields a purple aleurone but colorless scutellum. Moreover, the *R1-nj* marker system could also be used for twin-embryo kernel identification, i.e., owing to the deep-purple color pigmentation on the scutellum [42], the two germs of twin-embryo kernels were purple and easy to identify. Similarly, haploid twin-embryo kernels can be identified.

To study the relation between HIR and twin-embryo kernel frequency, two more inducer lines, namely CAU2 (HIR ~ 8%) and CAU^wx^ (developed by crossing CAU5 with a waxy inbred line; HIR ~ 5%), were introduced to pollinate ND5598, a hybrid with good seed set. For this study, TEKR was calculated with the formula TEKR (%) = (number of twin-embryo kernels/total number of kernels on the ear) × 100%. HIR was calculated according to Xu et al. [16].

### Observation and classification of twin-embryo kernels

Each of the twin-embryo kernels that we collected was given a code number and then scanned with a ScanMaker i800 (Microtek, Germany). Kernel type was classified based on morphology, size, and color of the two germs in the embryo. To visualize the embryos clearly, we carefully removed the endosperm from each kernel after soaking it in water at 45 °C for 20–28 h. The whole embryos were then isolated and examined under a stereomicroscope and photographed.

### Field performance of the twin-embryo plants

Twin-embryo kernels were germinated in an incubator. After germination, the morphology of the twin seedlings was investigated. Because the seedlings were too weak to grow outdoors, they were transplanted to a seedling nursery until the 4- to 5-leaf stage. The young twin plants were then transplanted to field. We focused on the agronomic traits of these twin plants, especially plant height, to assess possible differences between individuals.

### Ploidy of the twin-embryo plants

Several methods were used to assess the ploidy level of the twin-embryo plants. In seed stage, the pigmentation of *R1-nj* on scutellum was used for putative haploid twin-embryo kernel identification. Of the plants in the field, chromosome count was conducted in prophase or metaphase of male gametophyte meiosis I stage, which can determine the ploidy deirctly. To further confirm the ploidy of twin plants, we used flow cytometry, which can discriminate haploids, diploids, and aneuploids based on the DNA content of the plant cell nuclei [43]. Young leaf tissue was collected and then treated with cell lysis solution for 30 min within the 30-min after leaf collection. Nuclei extracted, and flow cytometry was used to determine DNA content as described by Kleiber et al. [44]. For all samples quantified with flow cytometry, young leaf samples of the typical haploid and diploid B73 were used for the control. Relative nuclear ploidy was determined according to the signal peak of B73 and of its corresponding haploid sample. Samples with the same peak as B73 were diploids, and samples with the same peak as the B73 haploids were haploids. Signal peaks for aneuploids were those neither with the same peak as haploids nor diploids [22]. Of 146 mature twin plants, 43 samples were taken from twin plants with large PHD and used to check the ploidy level by flow cytometry. These samples covered twin plants that grew poorly, putative haploids, and diploid plants based on their morphological characteristics. Compared with the diploids, haploid plants were shorter, had erect and narrow leaves, and were sterile.

### Molecular genetic differences between twin plants

Most of the twin plants in the field had similar phenotypes. However, a small proportion of plants differed substantially. To assess differences between the twin plants at the molecular level, we selected 50 pairs of twin plants that varied with respect to PHD for SSR analysis. DNA was extracted from leaf tissue using the CTAB method [45]. Thirty high-quality SSR markers (Additional file 4) with clear bands, i.e., after scanning the genome with 200 markers were used. We then examined the twin seedlings with 3072 SNP markers. A total of 30 individuals from 15 twin-embryo seeds in the three different groups were genotyped with the Illumina Golden-Gate SNP genotyping platform [46, 47]. These molecular markers were used to assess genetic differences between the twin plants.

## Additional files

Additional file 1: Frequency of twin-embryo kernels among 20 different inbred lines. The number of tested kernels, twin embryos and frequency of twin embryos among 20 different inbred lines after maternal haploid induction. (XLSX 9 kb)

Additional file 2: Frequency of twin-embryo kernels among some specific inbred lines. Frequency of twin-embryo kernels among some specific inbred lines induced by both CAUHOI and CAU5. (XLSX 10 kb)

Additional file 3: Information for 20 females. The detail information of these 20 females, including their names and heterotic groups respectively. (XLSX 8 kb)

Additional file 4: SSR markers used for genotyping twin plants. The SSR markers’ names and their genetic positions. (XLSX 9 kb)

## Abbreviations

DH: Doubled haploids
HI: Haploid induction
HIR: Haploid induction rate
PHD: Plant height difference
SNP: Single nucleotide polymorphism
SSR: Simple sequence repeat
TEKR: Twin-embryo kernel rate
